# Supplementary material for: Analysis of Protein-Protein Interactions for Intermolecular Bond Prediction
Source: Molecules. 2022 Sep 21;27(19):6178. doi: 10.3390/molecules27196178 (PMC9572624; doi:10.3390/molecules27196178)
Supplement: Supplementary file 1 [file molecules-27-06178-s001.zip › molecules-1772606-supplement.pdf]

SUPPLEMENTAL MATERIALS

Table S1. Bond predictions and validation by literature.

Protein/PDB	Bond Type/Distance	Interacting Amino Acid Pair	Shown by Literature
**1YI5**	**Ionic Bond**		
	5A	F ASP27 / B LYS34	N
	7.5A	F ARG33 / A GLU149	N
		F ARG33 / A ASP194	N
		F ARG36 / A ASP194	N
	10A	F ASP8 / A ARG148	N
		F ASP8 / A LYS180	N
		F ARG33 / A ASP85	N
		F ARG33 / A GLU190	N
		F LYS35 / B GLU163	N
		F LYS49 / B GLU163	N
		F ARG68 / B ASP108	N
		F ARG68 / B GLU110	N
	**Hydrogen Bond**	F ASP27 / A TYR185	Y
		F LYS35 / A SER186	Y
		F ASP27 / TYR185	
	**Salt Bridge**	F ASP27 / B LYS34	N
	**Missed**	None	
**4HQP**	**Ionic Bond**		
	5A	I ASP30 / A ARG182	Y
		I ARG36 / A ASP193	N
		I LYS38 / A GLU185	Y
	7.5A	I ARG25 / A GLU185	N
		I LYS38 / B GLU185	N
	10A	I ARG36 / A ASP87	N
		I ARG36 / A GLU151	N
		I LYS38 / B ASP160	N
		I GLU41 / A ARG182	N
		I HIS68 / A GLU185	Y
		I HIS68 / A GLU189	N
		I LYS70 / A GLU185	Y
	**Hydrogen Bond**	I VAL40 / A PHE183	Y
		I ASP30 / A TYR184	Y
		I LYS38 / A GLU185	Y
	**Salt Bridge**	I ASP30 / A ARG182	Y
	**Missed**	ARG36 / TYR91	Hydrogen Bond
		ARG36 / TRP145	Hydrogen Bond
		LYS38/GLU185	Salt Bridge
**2QC1**	**Ionic Bond**		
	5A	None	
	7.5A	A ARG36 / B ASP152	N
		A LYS52 / B GLU129	N
	10A	A ASP30 / B LYS145	N
		A ARG36 / B ASP89	N
		A ARG36 / B ASP200	N

Table S1. continued from previous page

Protein/PDB	Bond Type/Distance	Interacting Amino Acid Pair	Shown by Literature
		A GLU41 / B LYS145	N
		A GLU41 / B HIS186	N
	**Hydrogen Bond**	A ASP30 / B TYR190	Y
		A ARG36 / B CYS192	Y
		A ARG36 / B THR148	Y
		A ARG36 / B ARG149	Y
		A LYS38 / B SER191	Y
		A VAL40 / B PHE189	Y
		A HIS68 / B SER191	N
		A LYS70 / B CYS192	N
	**Salt Bridge**	None	
	**Missed**	None	
**Barnase/Barstar**	**Ionic Bond**		
	5A	A ARG59 / D GLU76	Y
		A ARG59 / D ASP35	Y
		A HIS102 / D ASP39	Y
	7.5A	A LYS27 / D GLU80	N
		A ARG59 / D GLU80	N
		A ARG59 / D ASP39	Y
		A GLU60 / D HIS17	N
		A ARG83 / D ASP39	Y
		A ARG87 / D ASP39	Y
	10A	A LYS27 / D ASP39	Y
		A LYS39 / D GLU46	N
		A LYS62 / D ASP35	N
		A HIS102 / D ASP35	Y
	**Hydrogen Bond**	A LYS27 / D THRE42	Y
		A ARG59 / D ASP35	Y
		A ARG59 / D GLU76	Y
		A GLU60 / D ASP35	N
		A GLU60 / D LEU34	Y
		A ARG83 / D TYR29	Y
		A ARG83 / D ASP39	Y
		A ARG83 / D GLY43	Y
		A ARG87 / D ASP39	Y
		A HIS102 / D ASN33	Y
		A HIS102 / D GLY31	Y
		A HIS102 / D ASP39	Y
	**Salt Bridge**	ARG59 / D GLU76	Y
	**Missed**	ARG83/ASP39	Hydrogen Bond + Electrostatic
		ARG87/ASP39	Hydrogen Bond + Electrostatic
		ASN84 / TYR29	Hydrogen Bond
**Rap1a/Raf**	**Ionic Bond**		
	5A	A GLU3 / B LYS65	Y
		A ASP33 / B LYS84	Y
		A GLU37 / B ARG67	Y
		A ASP38 / B ARG89	Y
	7.5A	A GLU37 / B ARG59	Y
		A GLU54 / B LYS65	N
		A GLU54 / B ARG67	N

Table S1. continued from previous page

Protein/PDB	Bond Type/Distance	Interacting Amino Acid Pair	Shown by Literature
	10A	A GLU30 / B LYS87	N
		A GLU37 / B ARG89	N
		A ASP38 / B ARG67	N
		A ASP38 / B LYS84	N
		A ASP57 / B LYS84	N
		A ASP57 / B ARG89	N
	**Hydrogen Bond**	A ASP33 / B LYS84	Y
		A GLU37 / B VAL69	Y
		A GLU37 / B ARG59	Y
		A ASP38 / B THR68	Y
		A ASP38 / B ARG89	Y
		A SER39 / B ARG67	Y
		A SER39 / B ARG89	Y
		A ARG41 / B ASN64	Y
	**Salt Bridge**	ASP33 / B LYS84,	Y
	**Missed**	ASP38 / ARG89	Salt Bridge
		GLU37/ARG67	Salt Bridge
		GLU37 / ARG59	Salt Bridge
		ARG41 / GLN 66	Hydrogen Bond
**1KC4**	**Ionic Bond**		
	5A	A LYS38 / B GLU188	Y
		A HIS68 / B GLU188	Y
	7.5A	A ARG25 / B GLU188	N
		A ARG36 / B GLU192	N
	10A	A ARG36 / B GLU184	N
		A ARG36 / B GLU188	Y
	**Hydrogen Bond**	A TRP28 / B TYR194	N
		A ARG36 / B GLU188	Y
	**Salt Bridge**	None	
	**Missed**	Arg36 / Phe186	Hydrogen Bond
		Arg36 / Tyr 194	Hydrogen Bond
**Smad2/Smad4**	**Ionic**	B chain with C chain	
	5A	B LYS340 / C GLU288	Y
		B ASP493 / C ARG321	Y
		B ASP493 / C ARG329	Y
	7.5A	B HIS317 / C ASP304	N
		B ASP493 / C ARG330	N
		B ASP494 / C ARG329	N
		B ASP494 / C ARG330	N
		B ARG496 / C GLU281	N
		B ARG496 / C GLU326	N
		B ARG497 / C GLU326	N
		B ASP537 / C ARG310	Y
		B HIS541 / C ASP300	N
		B ASP547 / C ARG330	N
		B ASP547 / C HIS331	N
	10A	B GLU337 / C ARG285	N
		B GLU337 / C HIS291	N
		B ASP494 / C ARG321	N
		B ARG502 / C GLU326	N

Table S1. **continued from previous page**

Protein/PDB	Bond Type/Distance	Interacting Amino Acid Pair	Shown by Literature
		B LYS519 / C GLU281	N
		B GLU526 / C ARG321	Y
		B HIS528 / C GLU288	N
		B HIS530 / C GLU288	N
		B HIS530 / C ASP304	N
		B ASP537 / C ARG329	N
		B HIS541 / C ASP304	N
	**Hbond**	B THR338 / C GLU288	N
		B LEU533 / C THR303	Y
		B ASP537 / C ARG310	Y
		B GLU526 / C SER317	Y
		B ASP493 / C ARG321	Y
	**Salt bridge**	B LYS340 / C GLU288	Y
		B ASP493 / C ARG321	Y
		B ASP493 / C ARG329	Y
	**Missed**	LYS340 / GLU288	Hydrogen Bond
		ASP332 / ASN320	Hydrogen Bond
		HIS528 / SER318	Hydrogen Bond
		GLN534 / ASP304	Hydrogen Bond
		ASP537 / THR303	Hydrogen Bond
		ASP537 / ASP304	Hydrogen Bond

Table S2. Free energy change from DiffBond prediction of bond removal compared to DiffBond prediction of no bond removal.

Total	Ionic Bond Prediction	Count	Avg ddG Normalized	STD	Confidence Interval	Avg ddG	CV
	Bond Broken	69	0.61	0.17	0.04	24.11	0.27
	Bond Intact	45	0.28	0.24	0.07	9.59	0.84
1BRS	Ionic Bond Prediction	Count	Avg ddG Normalized	STD	Confidence Interval	Avg ddG	CV
	Bond Broken	59	0.57	0.14	0.04	26.85	0.24
	Bond Intact	35	0.25	0.20	0.07	11.28	0.81
1C1Y	Ionic Bond Prediction	Count	Avg ddG Normalized	STD	Confidence Interval	Avg ddG	CV
	Bond Broken	9	0.80	0.16	0.10	7.52	0.20
	Bond Intact	7	0.49	0.30	0.23	1.40	0.62
Total	Hydrogen Bond	Count	Avg ddG Normalized	STD	Confidence	Avg ddG	CV
	Bond Broken	23	0.42	0.21	0.09	19.24	0.50
	Bond Intact	40	0.58	0.22	0.07	21.09	0.38
	Bond Formed	22	0.53	0.19	0.08	24.71	0.35
1BRS	Hydrogen Bond	Count	Avg ddG Normalized	STD	Confidence	Avg ddG	
	Bond Broken	22	0.43	0.21	0.09	19.83	0.50
	Bond Intact	32	0.52	0.20	0.07	24.48	0.38
	Bond Formed	22	0.53	0.19	0.08	24.71	0.35
1C1Y	Hydrogen Bond	Count	Avg ddG Normalized	STD	Confidence	Avg ddG	
	Bond Broken	1	0.32	0.00	n/a	6.17	0.00
	Bond Intact	8	0.80	0.15	0.10	7.51	0.18
	Bond Formed	0	n/a	n/a	n/a	n/a	n/a
Total	Salt Bridge	Count	Avg ddG Normalized	STD	Confidence Interval	Avg ddG	CV

Table S2. continued from previous page

Total	Ionic Bond Prediction	Count	Avg ddG Normalized	STD	Confidence Interval	Avg ddG	CV
	Bond Broken	46	0.56	0.22	0.06	22.02	0.40
	Bond Intact	17	0.45	0.22	0.10	15.60	0.49
1BRS	Salt Bridge	Count	Avg ddG Normalized	STD	Confidence Interval	Avg ddG	CV
	Bond Broken	40	0.51	0.20	0.06	24.03	0.39
	Bond Intact	14	0.40	0.21	0.11	18.46	0.52
1C1Y	Salt Bridge	Count	Avg ddG Normalized	STD	Confidence Interval	Avg ddG	CV
	Bond Broken	6	0.85	0.11	0.09	8.58	0.13
	Bond Intact	3	0.67	0.10	0.11	4.93	0.15

Table S3. Change in interface intersection volume calculated by VASP-E for each group of DiffBond prediction.

			Normalized k=1			Normalized k=5		
Total	Salt Bridge	Count	Volume Diff	Confidence	Avg volume	Volume Diff	Confidence	Avg volume
	Bond Broken	46	0.55	0.06	-791.41	0.63	0.05	-34.62
	Bond Intact	17	0.27	0.05	-139.74	0.37	0.05	-13.62
1BRS	Salt Bridge	Count	Volume Diff	Confidence	Avg volume	Volume Diff	Confidence	Avg volume
	Bond Broken	40	0.54	0.07	-779.23	0.68	0.04	-37.54
	Bond Intact	14	0.26	0.06	-92.14	0.38	0.06	-13.42
1C1Y	Salt Bridge	Count	Volume Diff	Confidence	Avg volume	Volume Diff	Confidence	Avg volume
	Bond Broken	6	0.60	0.15	-872.59	0.34	0.21	-15.16
	Bond Intact	3	0.32	0.15	-361.83	0.30	0.09	-14.54
Total	Salt Bridge	Count	Volume Diff	Confidence	Avg volume	Volume Diff	Confidence	Avg volume
	Bond Broken	46	0.55	0.06	-791.41	0.63	0.05	-34.62
	Bond Intact	17	0.27	0.05	-139.74	0.37	0.05	-13.62
1BRS	Salt Bridge	Count	Volume Diff	Confidence	Avg volume	Volume Diff	Confidence	Avg volume
	Bond Broken	40	0.54	0.07	-779.23	0.68	0.04	-37.54
	Bond Intact	14	0.26	0.06	-92.14	0.38	0.06	-13.42
1C1Y	Salt Bridge	Count	Volume Diff	Confidence	Avg volume	Volume Diff	Confidence	Avg volume
	Bond Broken	6	0.60	0.15	-872.59	0.34	0.21	-15.16
	Bond Intact	3	0.32	0.15	-361.83	0.30	0.09	-14.54
